# Surgical management of an odontogenic cutaneous sinus tract misdiagnosed for 4 years

**DOI:** 10.1002/ccr3.5333

**Published:** 2022-02-02

**Authors:** Hammouda Latifa, Touil Dorsaf, Kikly Amira, Jlassi Karim, Douki Nabiha

**Affiliations:** ^1^ 37966 Dental Faculty of Monastir Research Laboratory LR12ES11 University of Monastir Monastir Tunisia

**Keywords:** infection, misdiagnosis, odontogenic, radicular cyst, sinus tract

## Abstract

A cutaneous sinus tract of dental origin may easily be misdiagnosed and incorrectly treated. This paper reported a case of a 20‐year‐old male patient referred for a productive cutaneous sinus tract misdiagnosed by medical doctors for more than 4 years. The clinical and radiographic examinations confirmed the odontogenic origin related to a mandibular first right molar presenting an infected radicular cyst. Surgical treatment was performed leading to a significant healing of the sinus tract.

## Graphical abstract

Cutaneous sinus tracts of dental origin may easily be misdiagnosed. early and correct diagnosis of periapical root infections as well as their treatment can spare patients numerous other unnecessary treatments.

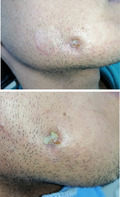

## 1 INTRODUCTION

Cutaneous sinus tracts of dental origin are relatively uncommon and are often initially misdiagnosed and inappropriately treated, due to their rarity and the absence of specific symptoms.^1^

This condition is defined as a pathologic canal leading from an enclosed area of inflammation or infection that opens to an epithelial surface of the face or the neck.^2^

Odontogenic cutaneous sinus tracts occur as a sequela of bacterial invasion of the dental pulp. Necrotic affected teeth exhibit apical periodontitis due to spread of infection into the periarticular area. The infection follows the path of least resistance between facial spaces, desiccate, and breakthrough the skin to form draining sinus tracts.^3^

A review of several reported cases showed that patients may undergo multiple unnecessary attempts of surgical excisions, drainage, biopsies, and long‐term use of antibiotics with no remission, due to improper diagnosis and the lack of treatment of the infectious dental origin.^4^, ^5^ Besides, in most cases, cutaneous sinus tracts of dental origin may not have any apparent dental symptoms and may progress over a long period of time without alarming the patient.

The aim of this paper was to present a case of a misdiagnosed cutaneous sinus tract related to an infected radicular cyst which was inappropriately treated for 4 years without any remission, and to highlight the importance of considering chronic dental infections as part of differential diagnosis for any orofacial skin lesion.

## 2 PATIENT AND OBSERVATION

A healthy 20‐year‐old male patient was referred by a general practitioner to the department of dentistry at the university hospital Sahloul for cutaneous productive sinus tract mimicking acne.

The patient reported a 4 years history of a drained and recurrent abscess in his right cheek that has previously been treated with different therapeutic procedure such as topical antibiotics and steroids (Bactrim, dermocort) prescribed by his dermatologist; however, no response was noted.

Besides, the patient did not report any history of dental pain or infection.

**FIGURE 1:**
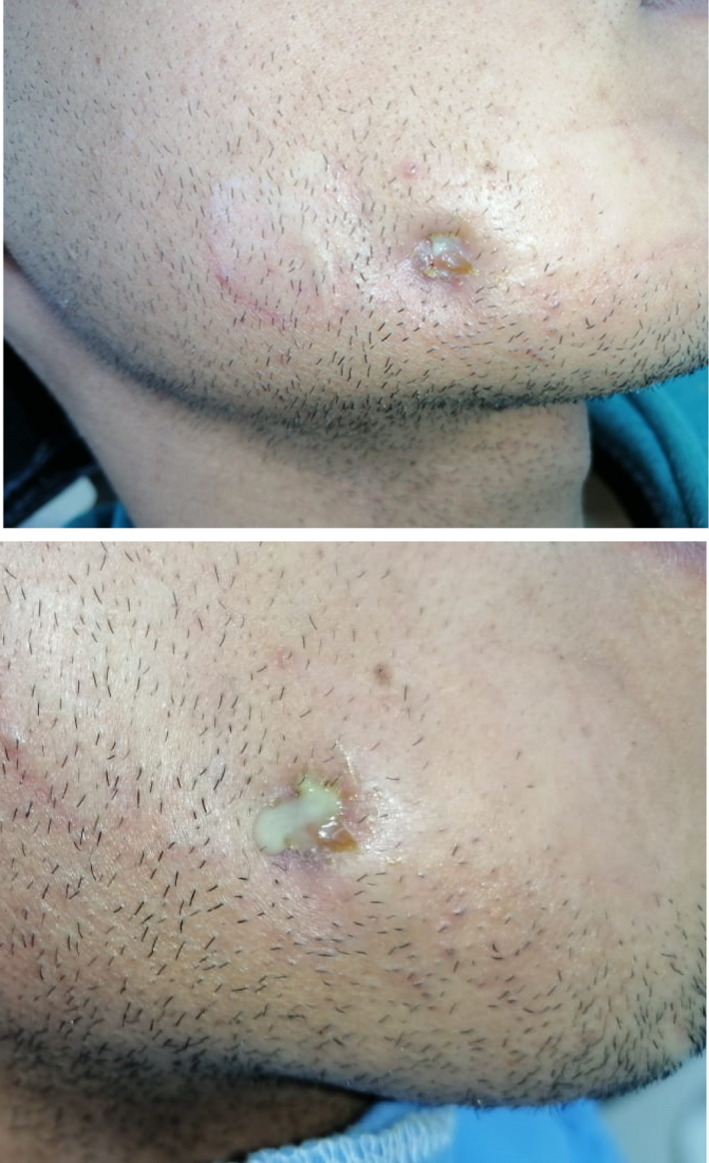
(A): Clinical view of the productive non‐retractile cutaneous fistula in the right cheek. (B): Cutaneous sinus tract with depression aspect, accompanied by a purulent discharge in the cheek region

The extra‐oral examination showed the persistence of a cutaneous fistula with a crusted appearance located on the lower part of right cheek measuring about 5 mm in diameter (Figure 1A). Gentle pressure on the surrounding tissue elicited a purulent discharge on the surface (Figure 1B).

Palpation showed the presence of a cord‐like tissue that linked to fistula to the mandibular vestibular bone.

**FIGURE 2:**
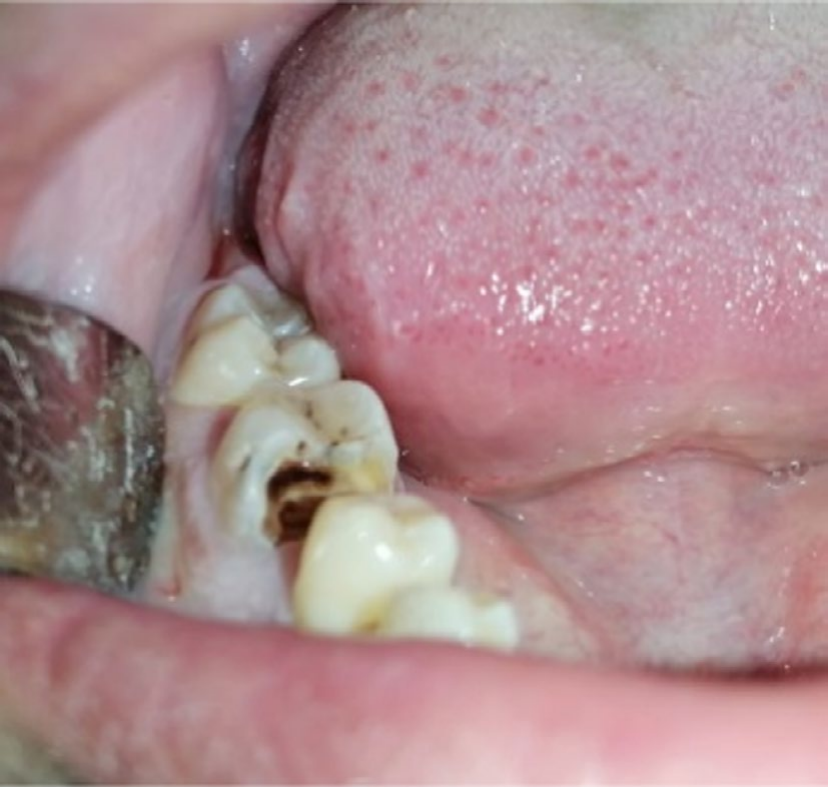
Intra oral view showing the presence of a deep carious lesion in the 46

The endobuccal examination revealed a poor oral hygiene and a decayed mandibular first molar (tooth 46) (Figure 2).

Pulp testing of the tooth 46 was negative while percussion, and periodontal probing revealed normal responses. No sign of mobility was present. Neighboring and contralateral teeth were also tested and were all within normal.

**FIGURE 3:**
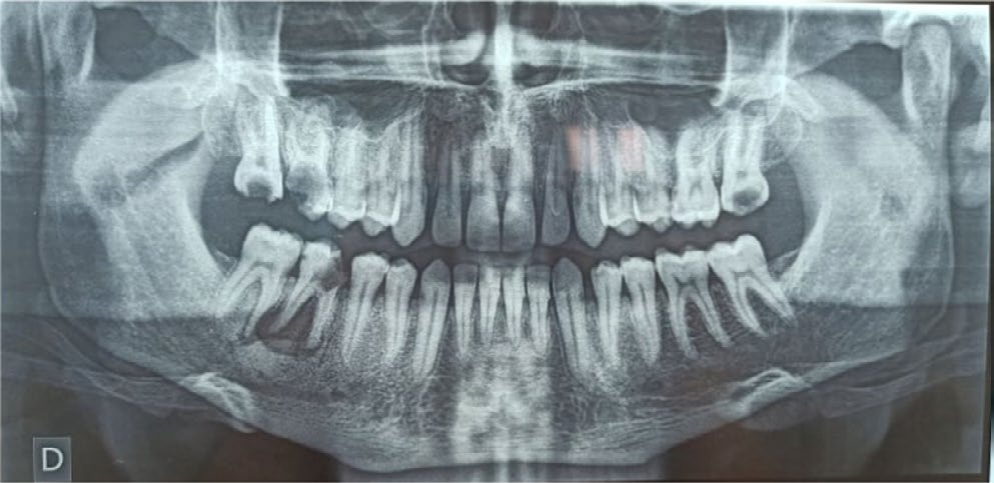
Panoramic radiograph revealed a deep carious lesion with exposed pulp on the tooth 46 and a large periapical radiolucency in relation with the two roots of 46

Panoramic radiograph revealed a large radiolucency of 2 cm diameter with a well‐defined cortical border in relation with the roots of the tooth 46 (Figure 3). A radio‐opaque lesion was also noticed in the same area and was associated with root resorption of the tooth 47.

**FIGURE 4:**
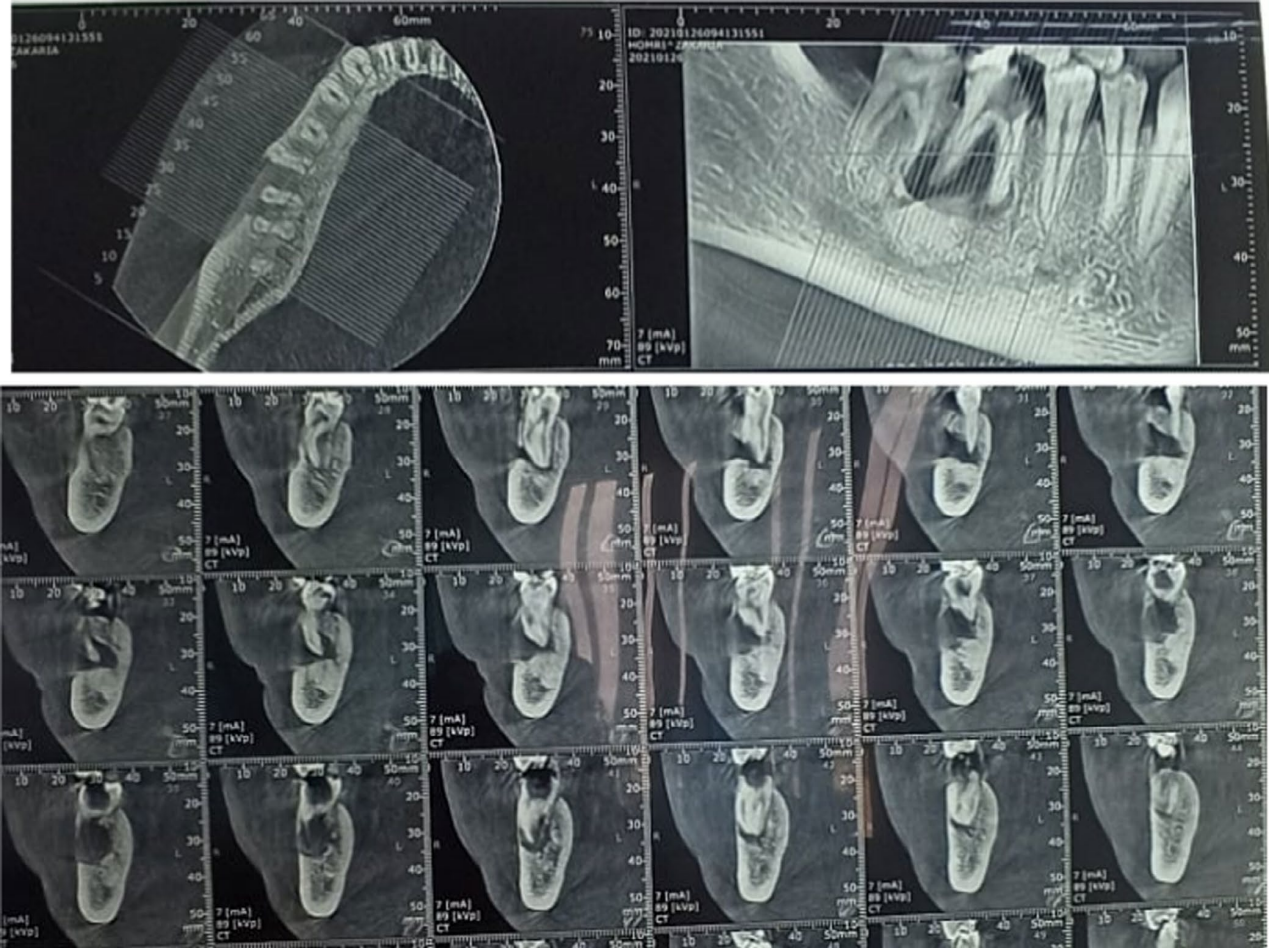
Axial and coronal cuts of a computed tomography (CT) scan confirmed the presence of periapical cystic images with a local perforation on the buccal alveolar table in front of #46 associated to a reactive bone sclerosis

Computed tomography (CT) scan was indicated, and the axial slices confirmed the periapical lesion and showed a mixed radiolucent‐radiopaque appearance of the lesion, located on the apices of the tooth 46. It also revealed a local perforation on the buccal alveolar table in front of the same tooth (Figure 4).

The initial diagnosis of infected radicular cyst associated with a cutaneous sinus tract was made. Differential diagnosis included infected cemento‐osseous dysplasia (COD) due to the radiographic appearance of the lesion.

**FIGURE 5:**
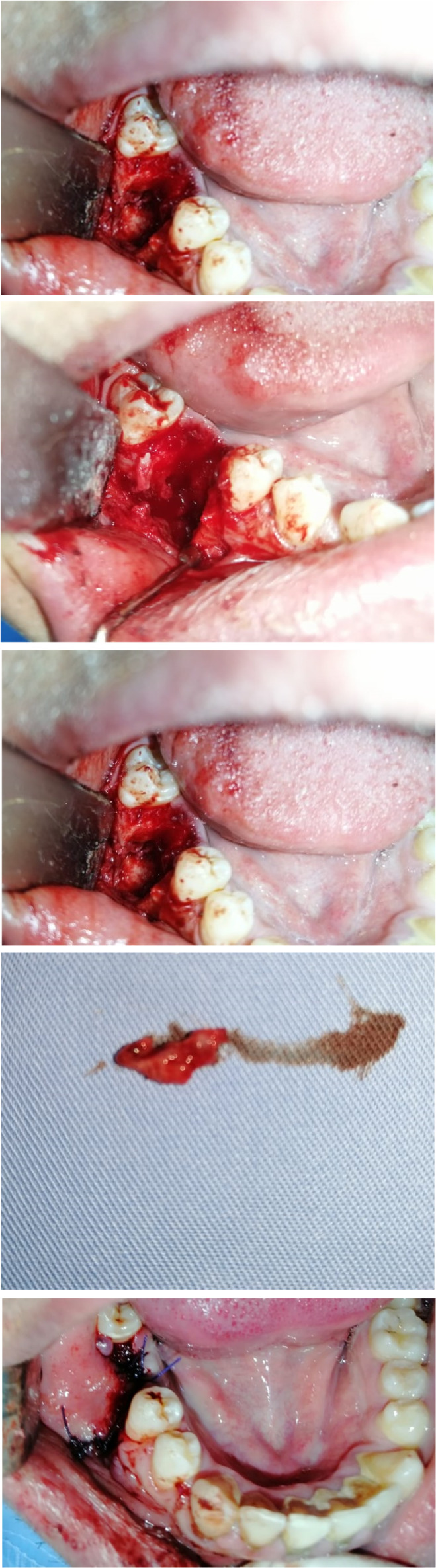
(A, B): Extraction of 46 and cyst enucleation An area of bone loss was visible after elimination of the cord‐like tract. (C): The cord‐like tract was excised from its origin to the skin. (D): wiew of the isolated sinus tract. (E): The sutures

The extraction of the tooth 46 and the radicular cyst enucleation under local anesthesia was indicated. Thus, a mucoperiosteal flap was elected and revealed a cord‐like tract attached to the vestibular bone in the periapical region. The tooth 46 was extracted followed by the excision of the radicular cyst (Figure 5A). A meticulous curettage of the alveolar site (Figure 5B) followed by the dissection and the excision of the cord‐like tract. The part of the tract attached to the bone was released (Figure 5C). after the excision of the whole sinus tract (Figure 5D), the skin was undermined to relax the affected area and restore normal facial contour. Sutures were placed (Figure 5E), and the patient was prescribed antibiotic therapy (amoxicillin–clavulanic acid) as well as antiseptic mouth wash and pain killers for 1 week.

As for the tooth 47, pulp testing was positive, thus only control sessions were scheduled and the endodontic treatment was postponed.

Histopathological examination of the lesion confirmed the diagnosis of odontogenic radicular cyst.

**FIGURE 6:**
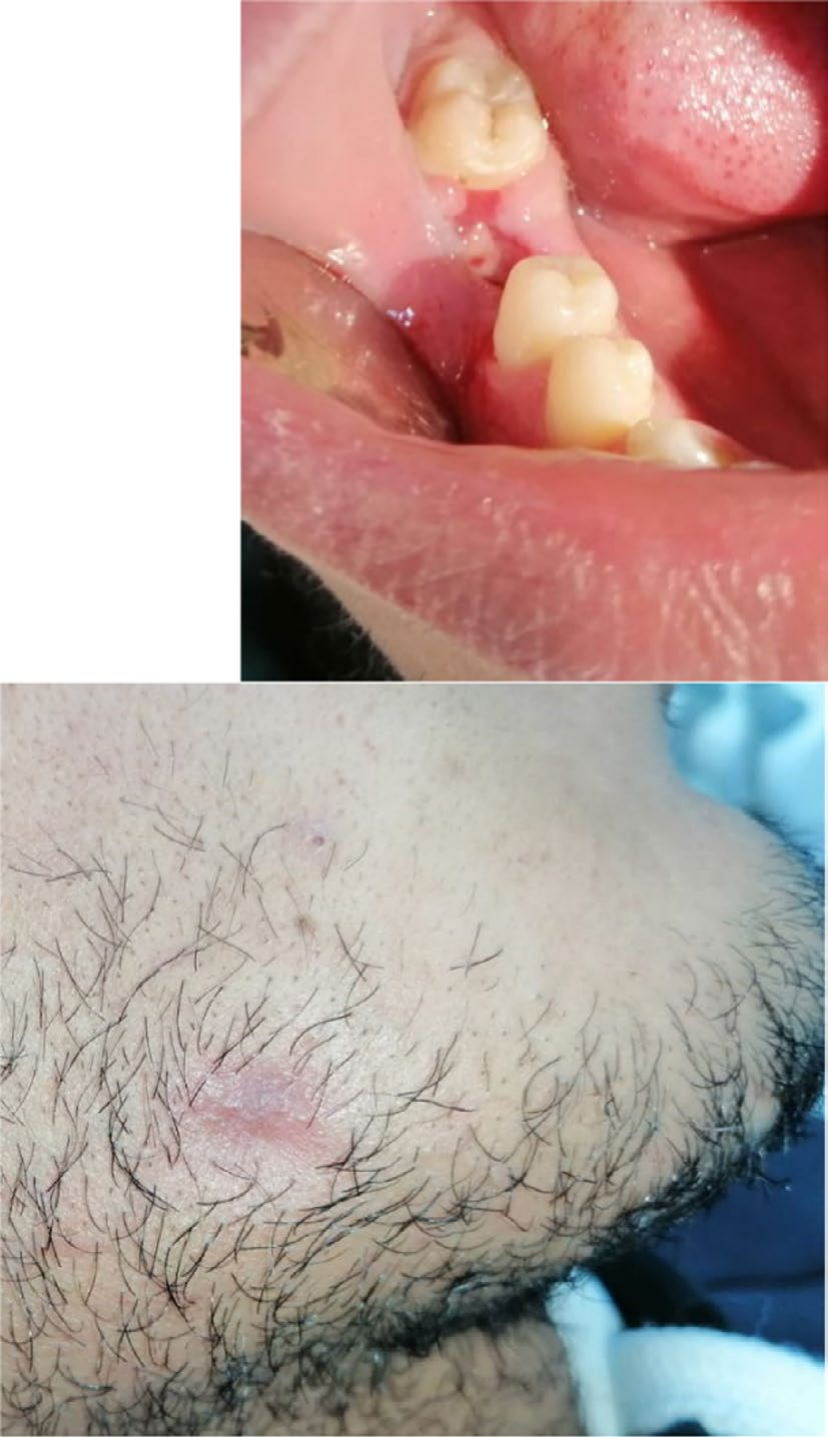
(A): Intraoral view,1 month after, revealed the healing of the alveolar site. (B): 1 month later: Significant healing of the sinus tract

Follow‐up was marked by the progressive healing to the cutaneous tract. At 1 month follow‐up, the lesion almost totally disappeared. (Figure 6A; Figure 6B).

## 3 DISCUSSION

Cutaneous fistula of dental origin are uncommon lesions, but have been well documented in the literature. However, misdiagnosis and inappropriate treatment often arise.^1^

Due to their location on the head‐and‐neck region, odontogenic cutaneous fistulas are the interest of several medical specialties.^6^ These tracts often have a clinical appearance similar to other skin lesions.^7^ The dermatologists and general practitioners are often consulted first.^6^ In this context, our patient consulted three general practitioners and two dermatologists, during four years, and was always prescribed different topical treatments including antibiotics and steroids but no remission was observed.

Besides, in most cases, cutaneous sinus tracts of dental origin may not have any apparent dental symptoms and may progress over a long period of time without alarming the patient.^7^

The odontogenic cutaneous sinus tract on the oro‐cervicofacial region often develops as a result of chronic apical lesion caused by pulp degeneration or necrosis. The apical infection may spread through the narrow space and then perforate the cortical alveolar bone. In soft tissue, the infection may spread through the path of least resistance between facial spaces and finally perforate a mucosal or cutaneous surface.^7^

In this fact, when diagnosing and treating sinus tracts of unknown etiology in the facial and cervical area, dermatologist or plastic surgeon should always refer patients to the dentists to eliminate a possible dental infection.^7^

Such diagnostic and therapeutic misadventures highlight the importance of collaboration between medical and dental practitioners in the management of patients with head‐and‐neck lesions.^8^

Early diagnosis and appropriate treatment are essential. A proper diagnosis should include medical history of the patient, inspection, and palpation of the cutaneous lesion.

Clinically, a cutaneous dental fistula has nonspecific skin manifestations and may resemble a pimple, ulcer, nodule, draining lesion, or indurated cystic area with purulent discharge^9^ which in the most cases are found on the chin and the cheek area but rarely in the nasal region.^10^

Samir et al described a classic cutaneous fistula with dental origin as an erythematous nodule of diameter up to 20 mm with or without drainage presenting skin retraction after healing.^11^

In this case, patient consulted for a suppurative cutaneous sinus tract with depression aspect in the right cheek with local alopecia of the whole area.

Besides, intraoral examination may reveal one or several decayed teeth, a healthy‐looking tooth with an intact crown but an endodontic infection, or injured tooth.^7^ With this regards, Chan et al reported an odontogenic cutaneous sinus tract caused by vertical root fracture.^12^ Calıskan et al. also reported a case of cutaneous sinus tract caused by a fractured crown.^13^

Pulp vitality test, percussion, and periodontal probing should be performed on the suspect tooth and adjacent teeth.^7^

Radiographic examinations, conventional or advanced imaging, should be indicated to identify a radiolucency at the periapical region of the suspected teeth.^8^

The indication of advanced 3D imaging is necessary, and patients should be evaluated using panoramic radiograph and cone‐beam computed tomography (CBCT) to evaluate the extend of the lesion and eventually confirm the causal tooth.^14^, ^15^

In the present case, the tooth 46 was severely decayed with an infra gingival and juxta‐osseous dental tissue destruction, pulp testing was negative, but surprisingly the patient did not report any episodes of pain or discomfort.

CBCT showed the existence of local perforation of the vestibular alveolar bone and a local bone sclerosis, which was also revealed by the conventional 2D radiography and which confirmed the chronic progressive evolution of the lesion.

A possible diagnosis of infected radicular cyst was made. Differential diagnosis included infected cemento‐osseous dysplasia (COD), due to the radiographic findings which revealed mixed radiolucent‐radiopaque appearance of the lesion, located on the apices of the tooth 46.

In some cases, the insertion of a probe or an endodontics gutta‐percha along the sinus tract is helpful for the ascertainment of the causal tooth,^10^but this is not usually possible with cutaneous sinus tracts—like in this reported case—due to the distance between the fistula orifice and the alveolar bone as well as the presence of multiple plans: mucosal, muscular, and cutaneous.^16^

The differential diagnosis includes traumatic lesions, fungal and bacterial infections, pyogenic and foreign body granulomas, basal cell carcinomas, local skin infections such as carbuncle and infected epidermoid cysts, chronic tuberculosis lesion, osteomyelitis, actinomycosis, and gumma of tertiary syphilis. Rare entities to be included in the differential diagnosis are developmental defects of thyroglossal duct origin or branchial cleft, salivary gland and duct fistula, dacryocystitis, and suppurative lymphadenitis.^10^, ^17^, ^18^

The treatment of odontogenic cutaneous sinus tracts requires the elimination of the infection origin. Systemic antibiotic therapy was reported to result in a temporary reduction of the drainage and an apparent healing.

However, the extraction, when indicated, or the conventional root‐canal treatment—when possible—is the treatment of choice.^14^

Antibiotics may be recommended as an adjunct to treatment in the setting of diabetes, immunosuppression, or systemic signs of infection such as fever, in fact systemic antibiotic administration is not indicated in patients with a cutaneous odontogenic sinus tract who have a competent immune system.^19^

After the eradication of the original source of infection, the sinus tract regularly disappears within 7–14 days after root‐canal treatment.^20^

In this case, the closure of sinus tract and the healing were seen 1 month later.

Root‐canal therapy is the treatment of choice if the tooth is restorable. Once the tooth is treated, the need for surgical excision is controversial. Some reports have indicated a complete excision of the sinus tract lining, while others have suggested that surgical treatment and antibiotic therapy are not necessary after dental treatment.^14^, ^21^

Root‐canal irrigation is a critical step on the success of root‐canal therapy because of the bactericidal action and elimination of necrotic tissue by the sodium hypochlorite. This step is usually followed by an intracanal medication with calcium hydroxide for its antimicrobial effects due to its high alkalinity; it has a destructive effect on cell membranes and protein structures, and stimulation of osseous repair^22^

Clinical and radiological follow‐up should be regularly performed to detect the absence of healing and the persistence of periapical lesion.

In this report, the extraction was indicated since that the tooth 46 was non‐restorable; thus, the extraction was associated with the enucleation of the radicular cyst and the simultaneous surgical excision of the sinus.

The antibiotic indication, in this case, is due to the heaviness of surgery following cyst enucleation.

As for the tooth 47, the radiographic investigations showed root resorption especially of the mesial one, but since pulp testing was positive, the endodontic treatment was postponed and control sessions were scheduled. The endodontic treatment should be performed as soon as any signs of infection or pulp necrosis are found.

Finally, in some cases, plastic surgery may be needed at a later step if healing results in cutaneous retraction are disappointing.^16^ Failure of a cutaneous sinus tract to heal after adequate root‐canal therapy or extraction requires further evaluation, microbiological sampling, and biopsy.^23^

## 4 CONCLUSION

This report highlights the importance of considering dental infection as a primary etiology in chronic cutaneous oro‐cervico‐facial sinus tracts.

Early correct diagnosis based on radiographic evidence of a periapical root infection and treatment of these lesions can spare patient numerous unnecessary and ineffective antibiotic therapy or plastic surgical treatment, reducing the possibility of further complications.

## CONFLICTS OF INTEREST

Authors declare no conflicts of interest.

## AUTHORS CONTRIBUTIONS

Latifa Hammouda and Dorsaf Touil have participated equally to the writing of the manuscript and have made the design, as well as the acquisition of data. Amira Kikly and Karim Jelassi have been involved in drafting the manuscript. Douki Nabiha contributed by revising critically for important intellectual content and has given final approval of the version to be published. All authors discussed the results and contributed to the final manuscript.

## CONSENT

A written consent has been obtained from the patient to write and to publish this report.

## Data Availability

The datasets generated during the current study are not publicly available but are available from the corresponding author on reasonable request.
